# Effect of shift-based scheduling on student learning, satisfaction and capacity in obstetrics and gynecology rotations

**DOI:** 10.5116/ijme.64b4.f880

**Published:** 2023-07-28

**Authors:** Erin Nelson

**Affiliations:** 1 Department of Obstetrics and Gynecology, University of Texas Health Science Center San Antonio, San Antonio, Texas, USA

**Keywords:** Clinical rotations, well-being, undergraduate medical education, shift-based schedule

## Abstract

**Objectives:**

Determine how a shift- based schedule to accommodate more students affects learning, performance, and satisfaction with the Obstetrics and Gynecology (OBG) rotation.

**Methods:**

The study was conducted among third year OBG medical students with a triangular convergent cross-sectional approach. A new shift-based schedule was implemented. After each rotation, an online survey was conducted using a convenience sampling. Student scores on the National Board of Medical Examiner (NBME) OBG subject exam were analyzed using paired t test. Survey data was analyzed using two sample t test. The relationship between survey responses and exam score findings were described. Data from shift-schedule students was compared to traditional schedule students from the prior academic year.

**Results:**

A statistically significant improvement was seen for average NBME score for shift-schedule students during the beginning portion (groups 1-3) of the academic year (M=80, SD=6.9) compared to traditional (M=75.7, SD=7.3) [t _(145)_ =3.69, p =.001]. A similar pattern was not seen in subsequent groups (groups 4-6). Shift-schedule students also showed a statistically significant improvement in their perception of learning (t _(183)_ =-2.54, p =.012). Parallel results were seen for belonging, manageable workload, time to study, and engaging meaningfully. Using this model, we increased rotation capacity from 24 to 30 students per group (20%).

**Conclusions:**

Shift based scheduling allows 20% increase in capacity. Exam scores and student learning outcomes were similar or better than traditional schedule controls.

## Introduction

Increasing numbers of learners and shortage of clinical sites require innovative strategies to address demand. Since 2002, Twenty-nine new accredited US medical schools have opened, along with 17 new schools of osteopathic medicine. Enrollment in U.S. medical schools has grown by 31% and combined with increases in enrollment at schools of osteopathic medicine, overall medical student enrollment is now 52% higher than in 2002.^1^

Increasing medical student numbers along with concomitant growth of physician assistant and nurse practitioner training programs (who also need clinical rotation sites) has compounded the need. This is not a uniquely American problem. Shortage of clinical training sites and clinical supervisors has been acknowledged around the world, particularly in Europe and Australia. Contributing factors are multiple including rising numbers of medical students, shorter hospital stays, more outpatient surgeries, and prehospitalization workups, and increasing demands on clinicians’ time.^2^

Medical education has experienced shifts in curricular organization, content, delivery, assessment, and the use of technology in response to the needs of our learners over the last decade.^3^ There are a variety of strategies, models, and frameworks^4^ that educators can use to design, develop, and deliver curricula and programs effectively and efficiently.

Additionally, there are measures and methods (i.e., written tests, clinical skills exams, faculty reports) that provide data to assess curriculum and student performance following curricular modifications.

One such change is shortening the traditional preclinical curriculum and allowing students to enter the clinical learning environment earlier in their training trajectory. This change results in challenges and downstream implications for medical schools and clinical partners alike. A report by Kraakevik and colleagues^5^ describes the experience of four different medical schools on how they managed the ‘bulge’ of clinical placements when students overlapped resulting from this transition between 2014-2019. Methods described included increasing the number of learners per site, creation of new electives, opening new clinical sites and having learners of different levels simultaneously in the clinical learning environment.

When assessing the effects of curriculum changes, student perception from their role as stakeholder provides a unique and important perspective. Makoul and colleagues^6^ underscore the value of gauging students’ perceptions regarding a variety of education goals.

Other studies have reported the value of gauging students’ perceptions regarding a variety of education goals and outcomes including well-being, learning activity, learning environment, and long-term effect.^7^^-^^9^

There are many factors that influence a student’s perception of his or her clinical rotation experience. A study by Gerbase and colleagues^10^ found that student opportunities to be involved in clinical practice to be the main factor influencing the overall perceived quality of most rotations. Additionally cited contributions included organization, students’ integration into rotation, improvement of clinical skills, supervision, and resident availability.

Several studies in the literature have evaluated the effect of shortening the clinical rotation length and its effect on student learning with varied outcomes.

Edwards and colleagues^11^ compared medical student performance on the obstetrics and gynecology national board subject examination scores between 2 different cohorts of students from 1994-1996 (8 week rotation versus 6 week rotation) demonstrating decreased NBME scores in students with the shorter rotation.

Reece and colleagues^12^ studied the effect of reducing each clinical rotation length by one week for one transitional year. In their cohort, there was no difference in performance on Step2 CK and CS or final rotation grades for shorter length students compared to historical controls. This is similar to the results of Strowd and colleagues^13^ who looked at student experience following an intervention that shortened clinical rotations by 20%. Student satisfaction and experience was similar and there were no significant differences in NBME, USMLE Step 2CK or OSCE scores compared to historical controls.

While many studies have investigated the effects of shortening overall clinical rotation length, none have evaluated a shift-based experience having each student spending fewer hours per day in the clinical learning environment to allow an increased capacity for a standard 6-week block.

The objective of this study was to investigate effects of a new shift- based scheduling in the OBG rotation on capacity, student learning and performance, and satisfaction.

We also wanted to compare outcomes for students who completed the rotation on the shift-based schedule to a group of control students from the year prior who had traditional shift schedules.

## Methods

### Study Design

A triangular convergent cross-sectional design was utilized to investigate the impact of shift-based scheduling for the OBG rotation. A new shift-based schedule was implemented at the beginning of the new academic year. Upon completion of each group, an online survey was conducted using a convenience sampling to assess student perception of the rotation, their learning and clinical experience. To design the survey tool and protocols, we engaged the Kirkpatrick^14^ model of curricular evaluation. This further provided us a framework to guide the analysis of outcomes and include aspects of learner reaction, learning, behavior change and results.

On the last day of the rotation, students take the National Board of Medical Examiner (NBME) OBG subject exam. These scores were collected at the same time as the survey and analyzed separately to provide an objective measure of student learning.

Survey data and Quantitative NBME exam scores were analyzed using t-tests. The relationship between survey and exam score findings was explored, and finally data collected from shift-schedule students was compared to the same data collected from non-shift-based schedule students from the prior academic year.

### Study Setting

The study setting was the University of Texas Health Science Center at San Antonio, Joe R. & Teresa Lozano Long School of Medicine. Following the preclinical curriculum, students embark on clinical rotations. There are eight “core” rotations: internal medicine, family medicine, surgery, pediatrics, emergency medicine, neurology, and obstetrics and gynecology. The OBG rotation is 6 weeks in length with students spending 3 weeks on obstetrics (OB) and 3 weeks on gynecology(G).

Prior to July 2018, students had a traditional 12-hour day schedule (5:00am-5:00pm M-Fri). In July 2018, a new clerkship schedule was implemented. This shift-based schedule was developed based on expert opinion after discussion with stakeholders including students, residents, faculty, medical educators. The main goals of the shift-based schedule were to provide a robust clinical experience with good coverage of areas within the specialty, mitigate long work hours and lack of predictability, maintain the ability of students to meet required encounters, and maintain or improve student satisfaction with the rotation experience.

Students worked fixed 8- or 9-hour shifts, equally distributed: 5:00am-1:30pm; 1:30pm-9:30pm and 9:30pm-7:00am (“night shift”). Over a week, students worked approximately 33% fewer hours compared to traditional 12-hour day schedules from the prior year.

### Study Participants and Sample size

Medical students completing their obstetrics and gynecology rotation between July of 2017 and January of 2019 comprised the study population. Students were excluded if they rotated off site, were not in classes of 2019 or 2020 or declined to participate. The study was reviewed by the Institutional Review Board (IRB) of the University of Texas Health Science Center at San Antonio and designated EXEMPT.

Sample Size Calculation: No sampling procedure was implemented since all eligible students were recruited for the study. The number of student eligible was 299.

**Figure 1:**
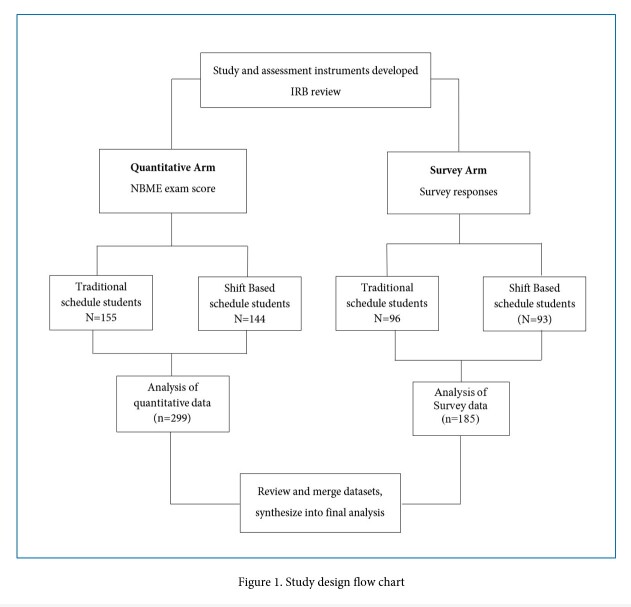
Study design flow chart

The outcome measures included Likert scores on survey (see below) which are reflective of student perception of their learning environment, clinical experience, team inclusion, well-being, and overall satisfaction (modified DREEM as described below). Quantitative outcomes were NBME exam scores (See Study design flowchart, Figure 1).

### Data Collection and Analysis

Survey Instrument: There are currently no validated survey instruments in the literature specifically addressing questions of work shift changes in the clinical learning environment, however our survey is modeled after the Dundee Ready Education Environment Measure (DREEM) published in 1997 by Roff and colleagues^15.^

The DREEM is a 50-item instrument developed to evaluate the educational environment in undergraduate medical education institutions and has been recommended as the most suitable tool available for this purpose.^16^ It includes five subscales which query student perceptions of learning, their teachers, atmosphere as well as academic and social self-perception. The DREEM has been translated into multiple languages and used globally.

Our survey instrument was developed to focus on student perception of the following domains:  workload, opportunities to interact with patients, ability to complete required clinical encounters, their sense of belonging and being meaningful participants in the clinical team, learning environment, study time, and National Board of Medical Examiner (NBME) subject exam preparation. These domains were selected to represent DREEM subscales of student perception of teaching, teachers, atmosphere, social self-perception, and self-performance. The tool was reviewed by recognized medical educators and their feedback was incorporated into the final draft of the survey. The instrument was piloted with a representative group of medical students and minimal adaptations were required. Content validity was supported through expert review of survey items, literature review and our piloting and review process for the instrument. The final instrument included 30 items which were scored using a 5-point Likert scale. The Likert scale was as follows; Never (1), rarely (2), sometimes (3), often (4) and always (5) for items 6-16 (domains of overall satisfaction, learning environment, team relations and well-being). Items 17-30, for domains of academic environment, engagement and inclusion utilized a Likert scale with Strongly disagree (1), Disagree (2), neither agree nor disagree (3), agree (4), and strongly agree (5). Reliability of the instrument was established using Cronbach alpha. (Alpha equals 0.85).

This was a closed online survey using the Qualtrics platform, accessible only via the student being logged into a university password protected account to avoid multiple entries per person. Students were recruited via e-mail invitations and the class Facebook page to participate. The first paragraph of the survey instrument included information about the study, name and contact information of the Principal Investigator, the IRB protocol review number, provision that participation was voluntary, and that responses had no effect on student grading for the clerkship. No compensation was offered.

Demographic information collected was limited to gender and year of graduation. 299 third- year medical students from the classes of 2019 and 2020 were invited to participate with a link sent via email upon completion of their OBG rotation. Surveys were collected between July of 2017 and January of 2019. The survey instrument contained 30 items which were distributed over 10 pages (3 items per page). A final completeness prompt allowing review of response was provided and a thank you message confirmed successful submission. Once a survey was completed it was not displayed a second time. Participation rate was 97.9% with 185 out of 189 students completing the survey.

### Analysis

Only completed surveys were entered into the study. Incomplete surveys were excluded from analysis (n=4). Survey data (survey responses and Likert scores) were compared between traditional and shift-schedule using two sample T test with statistical significance defined as a p value of <0.05. Average NBME exam score was compared between traditional and shift-schedule groups using paired t test with statistical significance defined as a p value of <0.05. The standard deviation on the NBME scoring was 7.3 for the traditional group and 6.5 for the shift-schedule group.

Analysis was performed using SAS (SAS Institute, Cary NC)

## Results

Using the shift based-schedule, we increased the number of students from 24 to 30 within the same 6-week rotation (20% expansion).

### Survey

Hundred and eighty-five (185) surveys were completed (response rate 49.3%), 50.8 % were male and 49.2% were female, 93 (59.2%) were from the class of 2019 and 92 (49.7%) were from the class of 2020.

A two sample t test on our survey instrument (modified DREEM scale) indicated that shift-based schedule students had a statistically significant improvement in their perception of learning (M=3.61, SD=1.07) compared to traditional schedule (M=3.23, SD=0.98) (t _(183)_ =-2.54, p =.012) The shift-based schedule students additionally demonstrated an increased sense of belonging (M=3.46, SD=1.07) compared to traditional schedule (M=2.97, SD=1.06) (t_(182)_=-3.11, p =.002). They also perceived having enough time to study at a higher level (M=4.17, SD=0.88) than traditional shift counterparts (M=3.52, SD=1.14) (t_(170)_=-4.27, p <.001) Shift- based students had a manageable workload (M=4.27, SD=0.72) at higher levels compared to traditional schedule students (M=3.83, SD=0.98) (t_(166)_=-3.47, p<.001). Shift-based students had higher levels of engagement (M=3.91, SD=0.86) compared to traditional schedule students (M=3.35, SD=1.00) (t _(180)_, p <.001) (Table 1).

### NBME score

A statistically significant improvement was seen for average NBME score in shift-schedule students during the beginning portion of the academic year (groups 1-3) with higher means (M=80, SD=6.9) for shift-based schedule students compared to traditional (M=75.7, SD=7.3) on paired t test [t _(145)_ =3.69, p =0.001]. A similar pattern was not seen in subsequent groups (groups 4-6). Post-hoc analysis was performed to investigate trends and it was determined that after group 3 (November), scores were similar between groups (Table 2).

## Discussion

The purpose of our study was to evaluate the impact of a new 8-hour shift-based schedule on learning outcomes and capacity on the OBG rotation and compare this to traditional 12-hour day historical controls.

**Table 1:** Traditional and shift-based schedule survey responses

Survey questions	Traditional (N = 96)	Shift-Based (N = 93)	t	p value^*^
	Mean (SD)	Mean (SD)		
Please rate your overall perception of the LEARNING ENVIRONMENT on the OBG clerkship	3.23 (0.98)	3.61 (1.07)	t_183_=-2.54	.012
I felt a sense of belonging during my time on the clerkship	2.97 (1.06)	3.46 (1.07)	t_182_=-3.11	.002
The workload on the clerkship is manageable	3.83 (0.98)	4.27 (0.72)	t_166_=-3.47	<.001
I had enough time to study on this rotation	3.52 (1.14)	4.17 (0.88)	t_170_=-4.27	<.001
I felt prepared for my NBME exam on this rotation	3.73 (1.12)	3.89 (1.01)	t_180_=-1.01	.31
The clerkship engages students as meaningful participants	3.35 (1.00)	3.91 (0.86)	t_180_=-4.08	<.001

^*^p < 0.05, 2 sample t-Test

Our data demonstrates an 8-hour shift- based schedule increased capacity by 20% while maintaining or improving student learning, performance on NBME exam and satisfaction with the learning experience. Shift based students spent approximately 33% less time in the hospital and clinics compared to their traditional 12-hour shift historical controls. Despite this, shift-based students’ satisfaction, and learning outcomes were similar or improved compared to 12-hour shift historical controls. Interestingly, students with the shift-based schedule anecdotally reported increased flexibility in balancing personal and professional responsibilities.

Review of the literature reveals no studies of shorter, shift- based OBG rotation schedules and their effect on student perceptions of their clinical experience, learning environment, or well-being.

Most studies have focused on shortening overall rotation length and examination score outcomes (NBME, Step 2 CS/CK, institutional clinical skills assessment) rather than the actual number of hours per day students spend in the clinical learning environment as we did.

**Table 2:** Average NBME scores by year and groups combined (post-hoc)

Variable		Intervention		p value^*^
		Pre	Post	
score of groups 1, 2, 3	N	75	72	
	Mean (SD)	75.7 (7.3)	80.0 (6.9)	.001
score of groups 4, 5, 6	N	80	72	
	Mean (SD)	80.2 (6.6)	79.6 (6.3)	.56

^*^p < 0.05, paired t-Test

A 2004 study by Myles^17^ on the effect of shortening OBGYN rotations from 8 to 6 weeks found the shorter rotation appeared to lower OBGYN final examination scores. Similarly, Edwards and colleagues^11^ demonstrated decreased NBME scores in students with shorter (6-week versus 8 week) rotation length (p<0.001).  Our findings for students spending fewer hours per day on the rotation demonstrating either no change or improvement NBME scores to contrast with both studies which found decreased NBME scores on shorter rotations. Edwards and colleagues also noted students who complete the rotation in the second half of the academic year scored higher compared to students in the first half (regardless of clerkship length) which we found in our cohort also.

In 2018, one US medical school (University of Michigan) reported that following a curricular modification which shortened all its required clinical rotations by 25%, there was no difference in NBME subject exam or year-end clinical skills exam scores. There was also no significant difference in student perception of rotation quality nor their perception of stress, well-being, or resiliency between cohorts.^18^ Our study findings of 8-hour shift students maintaining or improving learning outcomes and their perception of the rotation align with their results.

Strowd and colleagues^13^ looked at student experience following an intervention to shorten rotations by 20% noting similar student satisfaction and experience with the rotation and no significant differences in NBME, Step 2CK or institutional OSCE scores between cohorts.

Similarly, Reece and colleagues^12^ investigated reducing each rotation length by one week for one transitional year. They reported no difference compared to historical controls for performance on Step2 CK and CS, and similar final grades. Students had a positive perception of the change, however course directors voiced concern about educational experience and administrative burden engendered by the rapid change. Data from our study further supports the notion of both Strowd and Reece’s work that no adverse effect is seen with fewer hours on rotation.

A study by Talib and colleagues^19^ investigated incorporation of night hours into a pediatric rotation found they were able to increase capacity from 46 to 54 students (17.39% increase) while maintaining student satisfaction and cognitive performance19. Similar to Talib’s study we were able to support a 20% increase in learner number by incorporating 8-hour shifts of fixed length, which includes a week of night shifts, while maintaining student satisfaction and performance. Dolmans and colleagues^20^ from the Netherlands reported the effectiveness of clinical rotations depended on supervision and patient mix but not the number of students on the rotation. Perceived effectiveness was lowest for rotations with a limited patient mix and low clinical supervision and highest for rotations had a high patient mix and high levels of supervision. The study authors concluded high quality supervision guaranteed at least a sufficient score for the rotation regardless of patient mix. Results from our study supports these findings in that despite higher numbers of learners on rotation, given appropriate supervision and a diverse range of clinical experiences, student learning and performance are not negatively impacted.

### Strengths and Limitations

The main strength of the study is that it is the first to investigate using a shorter shift-based schedule for clinical rotations in medical education. The study included data from both survey responses as well as exam scores so outcomes could be evaluated from different perspectives. Another strength of our study was the high participation rate on the survey.

The study had several limitations. First is the study design, which uses historical controls as a comparison group rather than a true randomized control design with students from the same academic year. There may be confounding variables or differences between academic years we did not account for in the dataset. The study was performed at a single institution, potentially introducing bias, and student performance data from other clinical rotations was not collected for comparison. Although adapted from the Dundee Ready Education Environment Measure (DREEM), considered the most suitable tool to evaluate educational learning environments available, our survey was not previously validated.

Future directions for study include expansion of the shorter, shift-based schedule to other institutions and specialties to investigate whether outcomes are similar across a broader range of clinical learning experiences.

Additionally, evaluating perceptions of nursing staff, resident physicians and faculty on the shift model and its effect (if any) on patient care delivery may add important additional information and could be a focus of future studies.

Finally, we recommend studies evaluating whether faculty perceive interaction with students to be different with this model and what implications that may have on their teaching.

## Conclusions

Using shift-based scheduling model, we successfully increased OBG rotation capacity by 20%. Data from our study indicates the transition to a shorter, shift based (8-9 hours) clinical experience improved student perception of the learning environment, workload, and ability to study on the OBGYN rotation. This shorter shift-based schedule was associated with similar or improved outcomes in learning and student experience on the OBG rotation. Clerkship quality and clinical experiences were similar to traditional shift controls. Overall, students spent approximately 33% fewer hours in the clinical work environment. Shift based scheduling may allow students to better balance clinical, study and personal time. Shift based scheduling could have a major potential implication for medical education where clinical slots are at a premium.

### Acknowledgments

Zhu Wang PhD, Xuemei Song, Joel Michalek, PhD Stephanie Hernandez, MS and Jessica Perry, BS for statistical support. Veronica Riggs, MS4, and Keri Rowley, MS4 for assisting with survey piloting, recruitment of subjects.

### Conflict of Interest

The author declares that she has no conflict of interest.
